# Functional analysis of BARD1 missense variants in homology-directed repair and damage sensitivity

**DOI:** 10.1371/journal.pgen.1008049

**Published:** 2019-03-29

**Authors:** Aleksandra I. Adamovich, Tapahsama Banerjee, Margaret Wingo, Kathryn Duncan, Jie Ning, Fernanda Martins Rodrigues, Kuan-lin Huang, Cindy Lee, Feng Chen, Li Ding, Jeffrey D. Parvin

**Affiliations:** 1 Department of Biomedical Informatics, Ohio State University Comprehensive Cancer Center, Ohio State University, Columbus, Ohio, United States of America; 2 McDonnell Genome Institute, Washington University School of Medicine, Washington University in St. Louis, St. Louis, Missouri, United States of America; Cleveland Clinic Genomic Medicine Institute, UNITED STATES

## Abstract

The BARD1 protein, which heterodimerizes with BRCA1, is encoded by a known breast cancer susceptibility gene. While several *BARD1* variants have been identified as pathogenic, many more missense variants exist that do not occur frequently enough to assign a clinical risk. In this paper, whole exome sequencing of over 10,000 cancer samples from 33 cancer types identified from somatic mutations and loss of heterozygosity in tumors 76 potentially cancer-associated *BARD1* missense and truncation variants. These variants were tested in a functional assay for homology-directed repair (HDR), as HDR deficiencies have been shown to correlate with clinical pathogenicity for *BRCA1* variants. From these 76 variants, 4 in the ankyrin repeat domain and 5 in the BRCT domain were found to be non-functional in HDR. Two known benign variants were found to be functional in HDR, and three known pathogenic variants were non-functional, supporting the notion that the HDR assay can be used to predict the clinical risk of *BARD1* variants. The identification of HDR-deficient variants in the ankyrin repeat domain indicates there are DNA repair functions associated with this domain that have not been closely examined. In order to examine whether BARD1-associated loss of HDR function results in DNA damage sensitivity, cells expressing non-functional BARD1 variants were treated with ionizing radiation or cisplatin. These cells were found to be more sensitive to DNA damage, and variations in the residual HDR function of non-functional variants did not correlate with variations in sensitivity. These findings improve the understanding of BARD1 functional domains in DNA repair and support that this functional assay is useful for predicting the cancer association of *BARD1* variants.

## Author summary

*BARD1* is a breast cancer susceptibility gene encoding a protein that primarily interacts with BRCA1 in DNA repair. Although several *BARD1* variants are known to be pathogenic, many more variants do not occur frequently enough to assign a clinical risk. In this paper, we identified 76 potentially cancer-associated *BARD1* variants from analysis of over 10,000 tissue samples from people with cancer. It has previously been shown that if a BRCA1 variant cannot repair damaged DNA, then it is likely to cause cancer. We tested *BARD1* variants for DNA repair function and identified several non-functional variants that were localized in parts of the BARD1 protein not previously associated with DNA repair. Known benign BARD1 variants were found to be functional and known pathogenic variants were non-functional, showing that examining DNA repair function predicted variant pathogenicity. Cells expressing repair-defective BARD1 variants were also more sensitive to DNA damaging agents. These findings help us better understand how BARD1 is involved in DNA repair and show that examining the DNA repair function of BARD1 variants is useful for predicting their cancer risk.

## Introduction

Variants in *BRCA1* and *BRCA2* account for a plurality of hereditary breast and ovarian cancer (HBOC) cases, and are associated with risks of 50–85% for breast cancer and 15–40% for ovarian cancer [1–4]. BARD1 forms an obligate heterodimer with BRCA1, which functions as both an E3 ubiquitin ligase [5,6] and as a direct mediator of homologous recombination for the recruitment of RAD51 to the sites of DNA double-strand breaks [7–9]. Truncated *BARD1* variants have been identified in breast and ovarian cancers [10–12] and germline variants in the *BARD1* gene are associated with increased cancer risk [13]. Still, for both *BRCA1* and *BARD1*, the functional and clinical consequences are often unknown for sequence changes that replace the encoded amino acid residue.

Both *BRCA1* and *BARD1* are tested on clinical gene panels for breast and ovarian cancer susceptibility. Many *BRCA1* variants, as well as a few *BARD1* variants, have been determined to be clinically pathogenic. However, many more variants, which are generally missense substitutions, do not occur frequently enough in the population to assign a cancer risk and are classified as variants of uncertain significance (VUS). The ClinVar database [14] gathers information on pathogenic and benign variants, but most variants in its database are VUS. A gene panel testing 25 breast cancer-associated genes found 42% of all tests have findings of a VUS in one or more genes, indicating many people have such variants and there is a growing need for their classification [15]. Datasets such as the Cancer Genome Atlas (TCGA) gather information on missense variants, but are unable to be used for the accurate prediction of the cancer predisposition of a specific VUS. Assays examining homology-directed repair (HDR) function have demonstrated that known pathogenic *BRCA1* variants are non-functional in HDR, while benign variants are functional [16–19]. *BARD1* consists of an amino-terminal RING domain, three ankyrin repeat domains, and two carboxy-terminal BRCT domains [5,20]. Previous work in our lab has examined the HDR function of 29 *BARD1* variants, focusing on the RING and BRCT domains [21].

In this study, we identified 76 *BARD1* missense and truncation variants that were potentially cancer-associated from a large dataset containing exome-sequencing data on matched germline and tumor samples [19,22], and tested them for HDR function. Several HDR-deficient variants were identified in both the ankyrin repeat and BRCT domains. To examine the effects caused by loss of HDR function, cells expressing HDR-deficient BARD1 variants were treated with DNA damaging cisplatin or ionizing radiation. Cells expressing HDR-deficient variants were more sensitive to DNA damage and formed significantly fewer colonies than cells expressing wild-type BARD1. Although cells expressing HDR-deficient variants were more sensitive to damage than wild-type cells, quantitative variations in HDR deficiency did not correlate with differences in sensitivity to DNA damage agents. The results of this study reveal functional domains of BARD1 and suggest that the functional analysis of BARD1 HDR activity is predictive of breast and ovarian cancer risk.

## Results

### Identification of *BARD1* variants as potential cancer-associated loss of function variants

**Fig 1:**
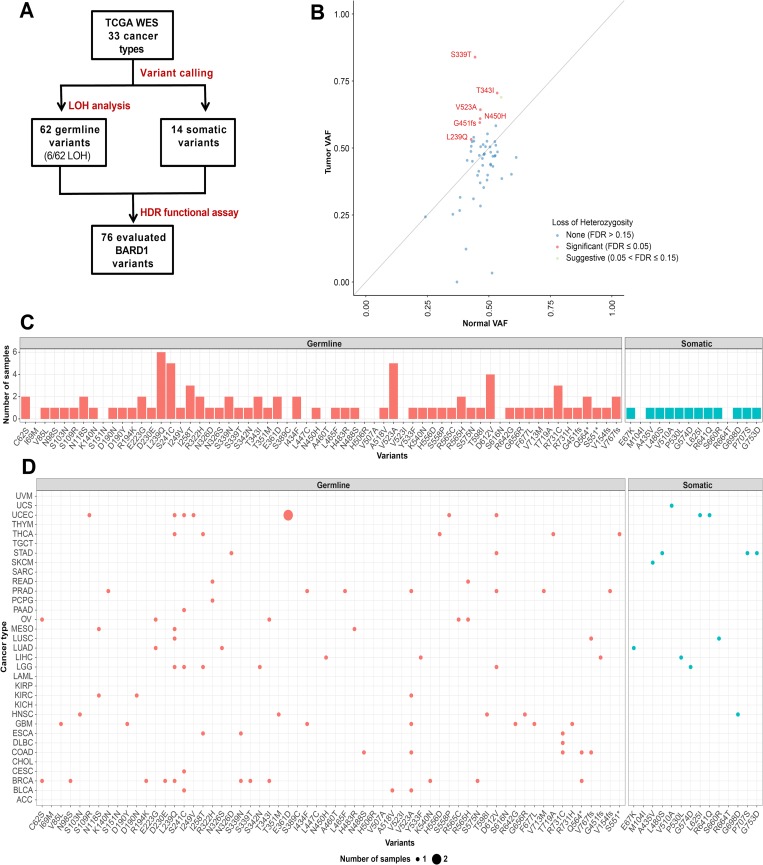
Selection of *BARD1* missense variants for functional analysis using sequencing data of cancer patient samples. (A) *BARD1* missense variants of interest were identified in a cohort of 4,034 samples from 12 cancer types and a larger set of 10,389 TCGA samples from 33 cancer types with whole exome sequencing [19,22]. (B) Identification of LOH in BARD1 through comparison of VAF in tumor and normal samples. Each dot depicts one variant. The diagonal line denotes neutral selection of the germline variant where the normal and tumor variant allele frequencies (VAFs) are identical. LOH was considered significant at False Discovery Rate (FDR) ≤ 0.05. (C) Number of samples containing each of the 76 *BARD1* variants in the 10,389 cohort [22]. (D) Number of samples affected by each *BARD1* variant for each of the 33 cancer types.

*BARD1* missense variants with potential cancer predisposition were identified in a set of 10,389 TCGA cancer samples from 33 cancer types using whole exome sequencing (Fig 1A) [19,22]. 62 rare germline variants and 14 somatic variants were found with variant calling. The variant allele frequency (VAF) and loss of heterozygosity (LOH) of germline variants were also examined to identify variants that could be functionally important. Six variants—S339T, T343I, V523A, N450H, G451fs and L239Q—were identified as having significantly higher LOH, indicating they had an increased likelihood of being pathogenic (Fig 1B). At the beginning of this study, variants were selected from a cohort of 4,034 samples [19,23] that later became part of a larger set of 10,389 samples [22,24]. Because of changes to selection criteria and data analysis, most, but not all, of the variants analyzed in this study were present in the larger data set, which was used to update the variant calling (Fig 1C). While several variants were not present in our newer data set, they are still likely present in the exome sequencing data. Analyzed variants were present in 24 of the 33 cancer types examined, not just breast or ovarian cancer, as might be predicted for a BRCA1 binding partner (Fig 1D).

### Analysis of *BARD1* variants in Homology-Directed Repair (HDR)

**Fig 2:**
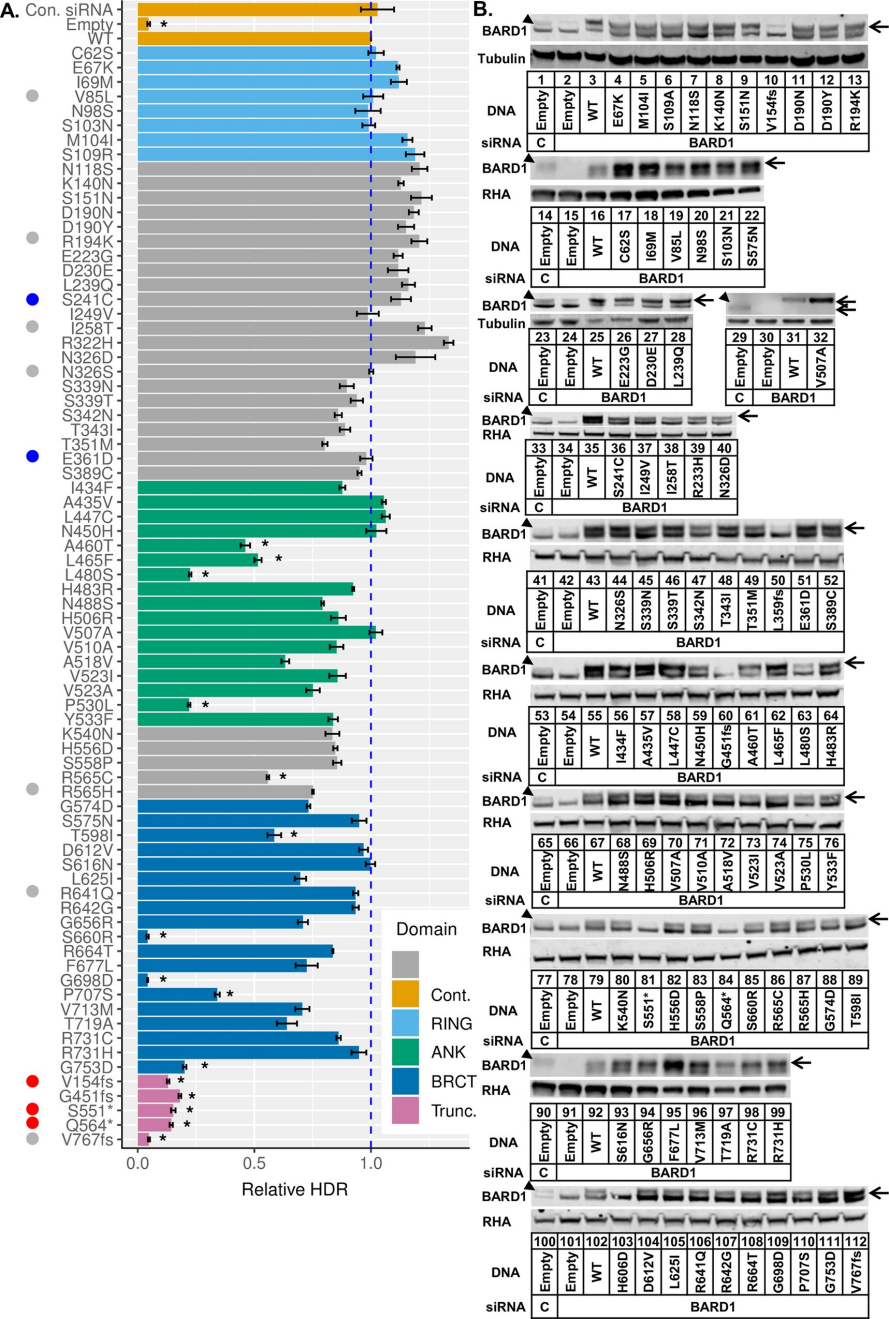
Functional analysis of *BARD1* variants. (A) 76 *BARD1* single missense substitutions were tested for function in the HDR assay. HeLa-DR cells [16] were treated with control siRNA (lane 1) or siRNA specific to the *BARD1* 3'-untranslated region (UTR) (lanes 2–80) and empty vector (lanes 1,2) or *BARD1* expression plasmid (lanes 3–80). Two positive controls were used: cells treated with empty vector and control siRNA (lane 1), and cells depleted of endogenous BARD1 with wild-type BARD1 rescue (lane 3). Cells treated with empty plasmid and *BARD1* 3'UTR siRNA were used as a negative control (lane 2). HDR function was characterized by the percentage of GFP-positive cells measured using flow cytometry. Results in each experiment (±S.E.M.) were normalized to the WT rescue (lane 3), which was set equal to 1. Results represent three independent transfections per *BARD1* plasmid. Variants that are benign and pathogenic according to ClinVar are labeled blue and red respectively. Variants with conflicting interpretations are labeled gray. HDR-deficient variants are marked by an asterisk and classified by having HDR function less than 0.6 and p < 0.01 when compared with endogenous BARD1 (control siRNA) using the Student’s *t*-test. (B) BARD1 variants tested in the HDR assay were examined for their expression relative to endogenous BARD1. Replicates were pooled together to examine BARD1 expression. The BARD1 protein is indicated with an arrow, as the BARD1-specific band migrated more slowly than a cross-contaminating band. The endogenously expressed BARD1 in control transfections (lanes 1, 14, 23, 29, 33, 41, 53, 65, 77, 90, 100) and the depleted BARD1 without rescue (lanes 2, 15, 24, 30, 34, 42, 54, 66, 78, 91, 101) can be compared with the expression of variant BARD1 proteins as indicated. A tagged *BARD1* V507A plasmid was used to confirm BARD1 expression (lanes 31 and 32, upper arrow) and migrated more slowly than endogenous BARD1 (lane 29, lower arrow). All missense variants had expression higher than the endogenously expressed BARD1.

76 *BARD1* missense variants, a majority of which were located in the ankyrin repeat and BRCT domains or between these domains, were tested for function in the homology-directed repair (HDR) assay (Fig 2A). For the HDR assay, a cell line that has two non-functional *GFP* coding sequences integrated into its DNA is used to examine DNA repair function. One of these GFP-encoding genes contains a recognition site for the rare-cutting restriction endonuclease I-SceI. When the I-SceI expression plasmid is transiently transfected into these cells, a double-strand break is made in one of the *GFP* sequences. If homology-directed repair uses the second *GFP* coding sequence as a template to repair across the double-strand break, then the encoded GFP is rendered functional [16,25]. We used a HeLa-derived cell clone called HeLa-DR, which has the GFP-encoding recombination substrate integrated at a single site. After transfection of the I-SceI expression plasmid, 10–20% of the cells were converted to GFP-positive [16]. Endogenous BARD1 expression was depleted in HeLa-DR cells by two rounds of transfection of a siRNA that targets the 3’-UTR of the *BARD1* mRNA. Simultaneously with the silencing of endogenous BARD1, BARD1 variants were expressed from transiently transfected plasmids that were resistant to the siRNA. Two days following the first transfection, the siRNA and plasmid were transfected again into the cells along with the plasmid that expresses the I-SceI endonuclease. Three days after the second transfection, the number of GFP-positive cells was determined using flow cytometry (S1 Table). Full HDR activity was observed under conditions of mock depletion of BARD1 by transfection with a control siRNA (Fig 2A, bar 1) and by depletion of BARD1 using the 3’-UTR targeted siRNA with rescue by transfection of a plasmid that expressed wild-type BARD1 (Fig 2A, bar 3). Cells depleted of BARD1 and transfected with an empty vector had a 25-fold decrease in HDR activity measured as the percentage of GFP-positive cells (Fig 2A, bar 2). We set the level of GFP expression following a double-strand break to a value of 1 relative to wild-type rescue (Fig 2A, bar 3) to facilitate comparison between experiments.

The 76 variants tested were from across the full coding sequence of *BARD1*. Variants whose HDR activity was lower than 0.6 and whose expression was greater than or equal to endogenous BARD1 were considered to be repair-deficient (Fig 2A and 2B). The eight variants located in the RING domain, as well the 22 in the region between the RING and ankyrin repeat domains, all had HDR activity similar to wild-type. Previous work in our lab [21] examined the HDR activity of 29 *BARD1* missense variants, including additional variants in the RING domain. We combined the current HDR results with the previously published results into a single table containing 105 variants (S1 Fig). In this previous work, the variants L44R, C53W, and C71Y in the RING domain were found to be defective in HDR due to defective binding to BRCA1. Surprisingly, in the current study, four of the 17 variants in the ankyrin domain, which has no known DNA repair function, were found to express full-length BARD1 and be defective in HDR. Variants A460T, L465F, L480S, and P530L had HDR activity lower than 0.6, which was significantly lower than cells expressing endogenous BARD1. The five variants located between the ankyrin repeat and BRCT domains were proficient in DNA repair with the exception of R565C, whose HDR activity was just below the cutoff of 0.6. Of the 19 missense variants tested in the BRCT domain, which is known to be involved in recruiting and retaining the BRCA1-BARD1 heterodimer to areas of DNA damage, five were found to be defective in HDR [26,27]. The variants S660R and G698D had HDR function comparable to cells transfected with empty vector. The variants T598I, P707S, and G753D had activity higher than empty vector but still significantly lower than endogenous BARD1. A larger fraction of residues conserved across several mammalian species were mutated in repair-deficient variants (9/10) than in functional ones (38/55) (S2 Fig). Five truncation variants were also tested, and all were about as equally defective as the empty vector in the HDR assay. Previous work has suggested that filtering using high LOH could be used to identify BRCA1 variants defective in HDR [19]. However, BARD1 variants that were found to have high LOH (Fig 1B) were all functional, with the exception of truncation variant G451fs.

Testing the HDR function of *BRCA1* variants has shown that, with the exception of variants that impact mRNA splicing, known pathogenic variants of *BRCA1* are HDR-defective, while known benign variants are not [16,17,19]. Similarly, the *BARD1* variants S241C and E361D, which have been found in patients with breast cancer and are benign according to ClinVar, are functional in HDR (Fig 2A, blue dots). Truncation variants V154fs, S551*, and Q564*, where the asterisk indicates a stop codon, are listed as pathogenic according to ClinVar and were non-functional in the HDR assay (Fig 2A, red dots). Several other variants that were tested have been identified in breast cancer patients and have conflicting interpretations of pathogenicity (Fig 2A, gray dots).

We tested whether the level of expression of any BARD1 variants could have affected their HDR activity. Expression of BARD1 variants was examined via immunoblot (Fig 2B). The relative expression of the endogenous BARD1 and siRNA-depleted BARD1 were shown (Fig 2B lanes 1, 2, 14, 15, 23, 24, 29, 30, 33, 34, 41, 42, 53, 54, 65, 66, 77, 78, 90, 91, 100,101). Though the expression levels of missense variants differed, they all expressed at higher levels than the endogenous BARD1. As an example, BARD1 L480S (lane 63) had lower expression than the plasmid encoded wild-type (lane 55), but both had more intensely labeled bands than the endogenously expressed BARD1 (lane 53). The variant BARD1 H606D is present in the immunoblots in Fig 2B (lane 103), but is not listed in Fig 2A because full length protein was not detected, and it was found to contain a nonsense mutation at codon 125. Similarly, BARD1 L359fs is present in Fig 2B (lane 50) and S3 Fig (lanes 5, 14) but is not listed in Fig 2A because it was a miss-call during variant selection. Frameshift and nonsense codon variants (Fig 2B lanes 10, 60, 81, 84, 112) lacked full length BARD1. Truncation variants G451fs, S551*, Q564* and V767fs expressed truncated BARD1, while variant protein V154fs was not detected (S3 Fig). We infer that repair defects observed in truncation variants with poor protein expression were due to the absence of protein instead of expressed, non-functional variant protein. We conclude that for the missense variants, a low level of HDR activity was not due to low expression of the BARD1 protein.

### Comparison of BARD1 HDR activity with sensitivity to DNA damage by ionizing radiation and cisplatin

**Fig 3:**
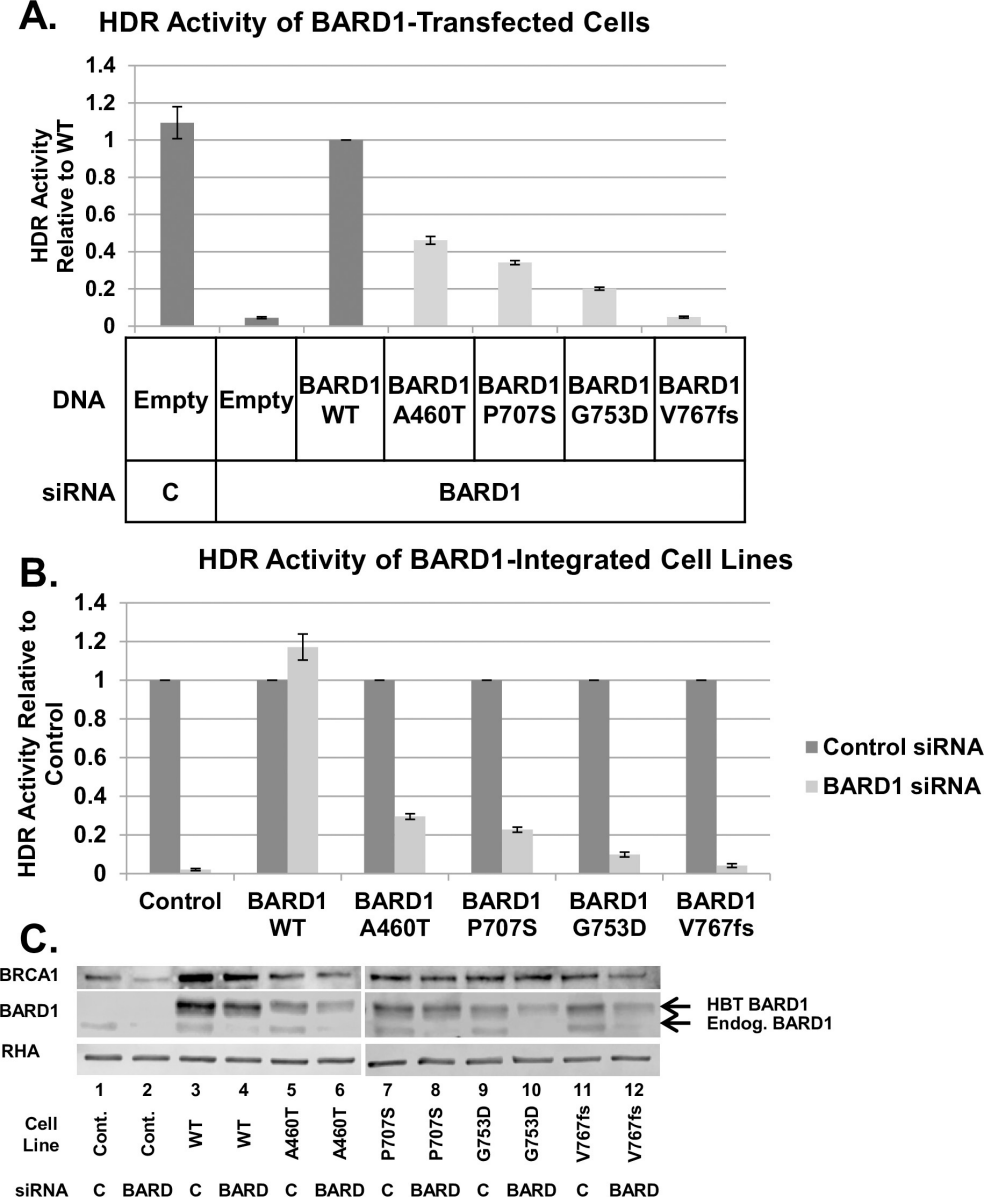
HBT-tagged BARD1 variants expressed from a single FRT site function similarly to transiently transfected variants in HDR. (A) Of the 76 *BARD1* variants examined for HDR functionality (Fig 2A), BARD1 A460T, P707S, G753D, and V767fs were selected for further study based on their range of HDR activity. (B) BARD1 variants integrated into a single FRT site in HeLa-DR-FRT/TR cells [29] functioned similarly to the same variants expressed in HeLa-DR cells by transient transfection. HBT-tagged BARD1 WT and variants were integrated into HeLa-DR-FRT/TR cells via the FRT site for consistent single-site expression. Cells were treated with control or *BARD1* 3'UTR siRNA and examined via the HDR assay. Unintegrated HeLa-DR-FRT/TR cells were used as a control. For comparison, results from transiently transfected cells were selected from Fig 2 (Fig 3A), and the results from expressing the integrated BARD1 variants are shown. While HDR activity was lower in cells containing integrated BARD1 as compared with transiently expressed BARD1, the integrated variants exhibited the same trend as the transiently expressed variants—BARD1 A460T was the most proficient, followed by BARD1 P707S, G753D, and V767fs. (C) Endogenous BARD1 was knocked down in treated stable cell lines, and BRCA1 and BARD1 expression were examined. HBT-tagged BARD1 variants migrated more slowly on electrophoresis gels than endogenous BARD1, as indicated by the upper (HBT-tagged) and lower (endogenous) arrows. HBT BARD1 was unaffected by the 3'UTR-targeted siRNA, while endogenous BARD1 was depleted. All of the BARD1 variants expressed at higher levels than the endogenously expressed BARD1 (*middle*). BRCA1 protein levels were not affected in variant-integrated cells (*top*).

BARD1 variants A460T, P707S, G753D, and V767fs were selected for further analysis, as they covered a range of HDR activities below 0.6 when transiently expressed. The selected variants and wild-type *BARD1* were tagged with the His-Biotin-Tobacco Etch Virus (HBT) tag [28] and integrated into the FRT site of a HeLa-DR derivative cell line called HeLa-DR-FRT/TR [29]. The advantage of these FRT site-containing cells was that the *BARD1* gene was stably expressed from a single site and should have consistent levels of expression. We tested the stably expressed variants in the HDR assay to confirm repair proficiency was the same as the transiently expressed variants. The HDR activities of these variants from Fig 2 are shown in isolation (Fig 3A). The same repair trends were observed in both transiently expressed and stably expressed BARD1 variants (Fig 3A and 3B). BARD1 A460T-integrated cells had the most residual repair activity among these variants, and cells integrated with BARD1 P707S, G753D and V767fs had decreasing levels of repair proficiency respectively. Expression of the integrated BARD1 variants was also greater than or equal to that of endogenous BARD1 (Fig 3C). BRCA1 expression in variant-integrated cell lines was similar in cells expressing endogenous BARD1 and the various defective BARD1 variants (Fig 3C). Thus, none of the changes in HDR observed with these BARD1 variants were attributable to changes in BRCA1 expression.

**Fig 4:**
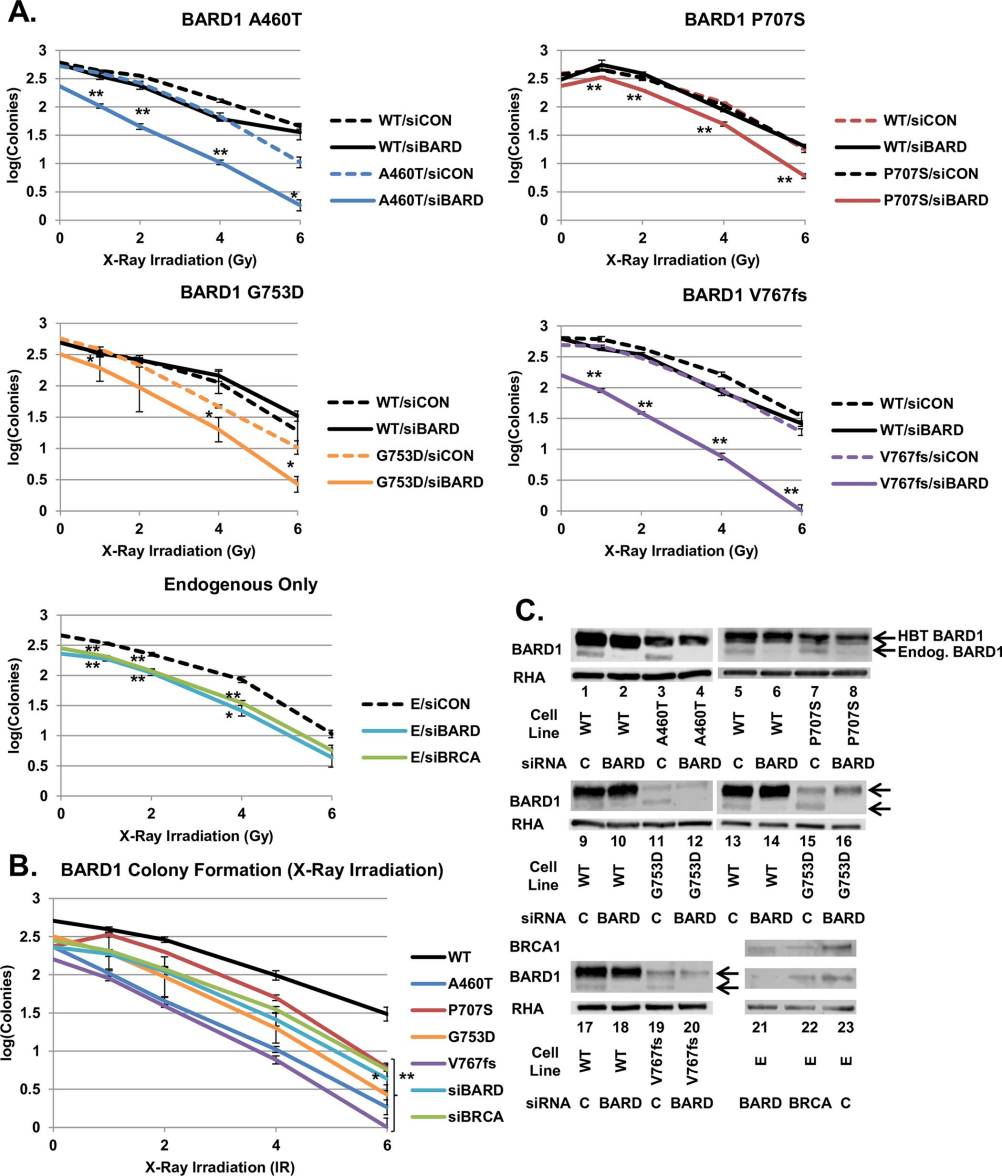
Sensitivity of *BARD1* variants to ionizing radiation. (A) Endogenous-only cells and cells expressing BARD1 WT, A460T, P707S, G753D, and V767fs were treated with control, *BARD1* 3'UTR or *BRCA1* 3’UTR siRNA and X-ray irradiated at doses of 1, 2, 4 and 6 Gy. Colonies were counted after 12 days of growth followed by staining with crystal violet. Results in each experiment (±S.E.M.) were carried out in triplicate and converted to logarithmic scale. In each plot, the dashed line indicates control siRNA (siCON), and the solid line indicates depletion with the *BARD1* (siBARD) or *BRCA1* (siBRCA) 3'UTR-targeted siRNA. The Student’s *t*-test was done to examine the growth of variant and endogenous-only cell lines treated with *BARD1* or *BRCA1* siRNA relative to variant and endogenous-only cells treated with control siRNA (indicated by asterisks; * = p < 0.05, ** = p < 0.01). Cells expressing the four BARD1 variants, as well as endogenous-only cells depleted of BARD1 and BRCA1, were significantly different from variant and endogenous-only cells treated with control siRNA across most irradiation doses. (B) Results from panel A are shown for the BARD1 WT and variant cell lines treated with *BARD1* 3'UTR siRNA and endogenous-only cells treated with *BARD1* or *BRCA1* 3’UTR siRNA. A Student’s *t*-test was done to compare the colony formation of BARD1 variant and BRCA1 or BARD1-depleted cell lines to BARD1 WT cells (indicated by asterisks). Colony counts of variants and BRCA1 or BARD1-depleted cell lines were significantly different (p < 0.01) from WT at all concentrations except siBARD 6 Gy (p < 0.05) and P707S 1 Gy, G753D 1 Gy, and G753D 2 Gy (p > 0.05). (C) An examination of the expression of endogenous BARD1 and BRCA1 in treated BARD1 variant-expressing and endogenous-only cell lines. Cells treated with *BARD1* 3'UTR siRNA had depleted endogenous BARD1 expression, while HBT BARD1 was unaffected. Cells treated with *BRCA1* 3’UTR siRNA showed depletion of endogenous BRCA1 and BARD1 expression. In all cases, the band representing the expression of the variant HBT BARD1 was denser than the faster migrating band from the endogenous BARD1 protein.

**Fig 5:**
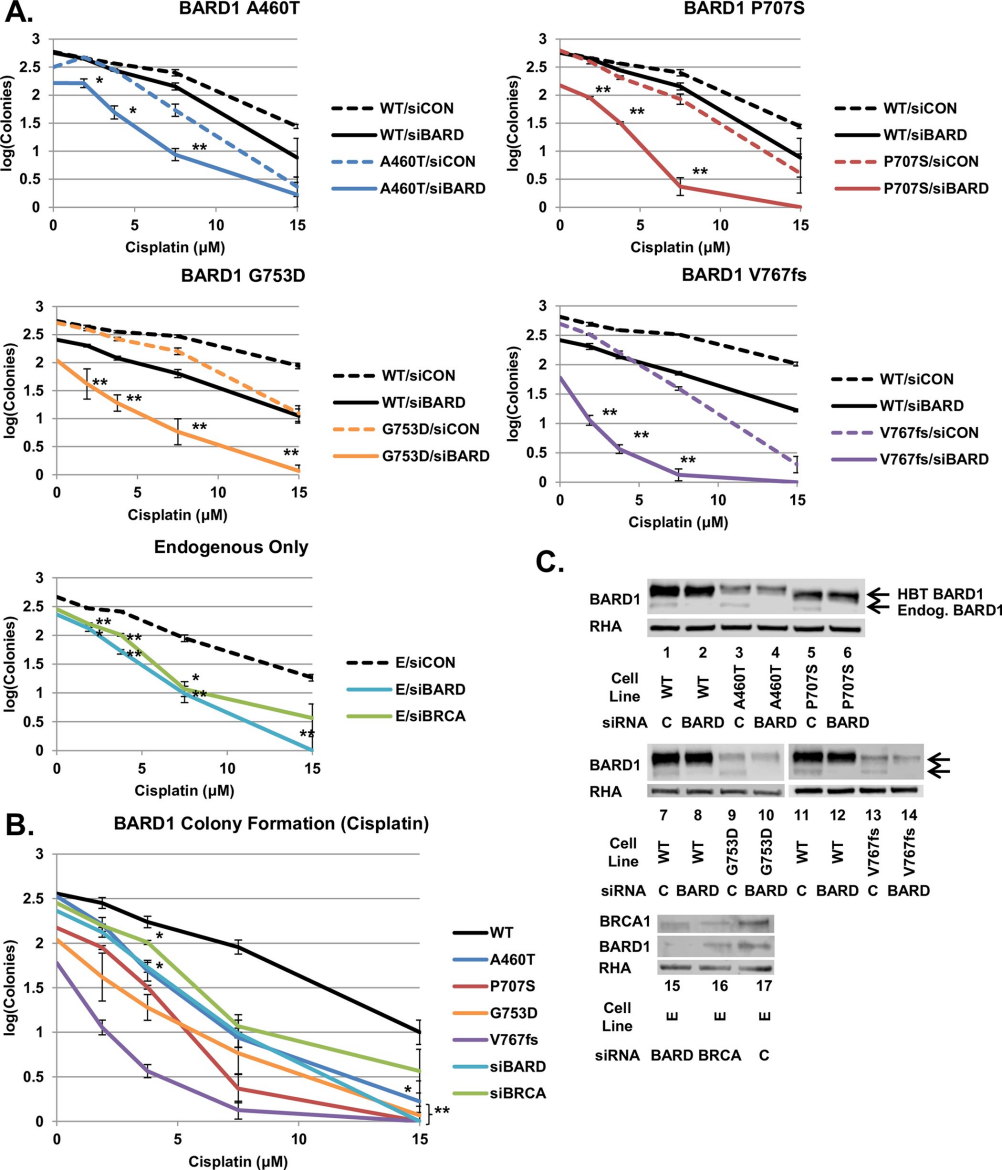
Sensitivity of *BARD1* variants to cisplatin. (A) Cells expressing BARD1 WT, A460T, P707S, G753D, and V767fs cells, as well as endogenous-only cells, were treated with control, *BARD1* 3'UTR or *BRCA1* 3’UTR siRNA and treated with cisplatin at concentrations of 1.875, 3.75, 7.5, and 15 μM. Colonies were counted after 12 days of growth followed by staining with crystal violet. Results in each experiment were done in triplicate and converted to logarithmic scale (±S.E.M.). In each plot, the dashed line indicates control siRNA, and the solid line indicates depletion with the *BARD1* or *BRCA1* 3'UTR-targeted siRNA. The Student’s *t*-test was done to examine the growth of variant and endogenous-only cell lines treated with BARD1 or BRCA1 siRNA relative to variant and endogenous-only cells treated with control siRNA (indicated by asterisks; * = p < 0.05, ** = p < 0.01). Cells expressing the four BARD1 variants, as well as endogenous-only cells depleted of BRCA1 and BARD1, were significantly different from variant and endogenous-only cells treated with control siRNA across most cisplatin concentrations. (B) Results from panel A are shown for BARD1 WT, variant and endogenous-only cell lines treated with *BARD1* or *BRCA1* 3'UTR siRNA. A Student’s *t*-test was done to compare the colony formation of BARD1 variant cell lines, as well as endogenous-only cells depleted of BRCA1 or BARD1, to BARD1 WT cells. Colony counts from variants and endogenous-only cells were significantly different (p < 0.01) from BARD1 WT at most concentrations, excepting A460T 3.75 μM, siBRCA1 3.75 μM and A460T 15 μM (p < 0.05) and A460T 1.875 μM and siBRCA 15 μM (p > 0.05). (C) An examination of endogenous BARD1 and BRCA1 expression in treated BARD1-expressing and control cell lines. Cells treated with *BARD1* 3'UTR siRNA had depleted endogenous BARD1 expression, while HBT BARD1 was unaffected. Cells treated with *BRCA1* 3’UTR siRNA showed depletion of endogenous BRCA1 and BARD1 expression. The band representing the expression of variant HBT BARD1 was denser than the endogenous BARD1 protein band in all cases.

We examined whether the quantitative loss of HDR proficiency correlated with the sensitivity of cells to extrinsic DNA damage. Clonogenic cell sensitivity assays were performed on HeLa-DR-FRT/TR cells expressing integrated BARD1 wild-type and variants, as well as endogenous-only unintegrated cells. Cells were depleted of endogenous BARD1 or BRCA1 and subjected to ionizing radiation (IR) (Fig 4) or cisplatin (Fig 5). Depletion of BARD1 or BRCA1 from the endogenous-only cells (E/siBARD and E/siBRCA) examined the effect of a non-rescued HDR defect on sensitivity to IR and provided a baseline for DNA damage sensitivity (Fig 4A, bottom). E/siBARD and E/siBRCA cells formed significantly fewer colonies after IR than control cells (E/siCON). BARD1 variant-integrated cells depleted of endogenous BARD1 (Variant/siBARD) all formed significantly fewer colonies following IR than the same cells treated with control siRNA (Variant/siCON) (Fig 4A, top). For ease of comparison, we included results from Variant/siBARD, E/siBARD, E/siBRCA and WT/siBARD cells on one graph (Fig 4B). Immunoblots were done to confirm knockdown of endogenous BARD1 (Fig 4C). While expression of each BARD1 variant differed, it was still greater than or equal to that of endogenous BARD1.

E/siBARD, E/siBRCA and Variant/siBARD cells formed significantly fewer colonies than WT/siBARD cells at most irradiation concentrations (Fig 4B). It was expected that increased HDR deficiency would result in increased sensitivity to DNA damage agents. For example, we expected that cells expressing BARD1 V767fs, the most HDR-deficient variant, would form the least number of colonies. Cells expressing BARD1 A460T, the variant with the most residual HDR activity, were expected to have the largest number of colonies compared with the other variants. Interestingly, this was not the trend observed in the results. Instead, HDR-defective BARD1 variants were all equally sensitive to IR, and as sensitive as non-rescued cells depleted of BARD1 or BRCA1. Similarly, all four BARD1 variants were more sensitive than wild-type to treatment with cisplatin (Fig 5A). Variant/siBARD, E/siBARD and E/siBRCA cells formed significantly fewer colonies than Variant/siCON and E/siCON cells. Variant/siBARD, E/siBARD and E/siBRCA cells also formed significantly fewer colonies than WT/siBARD cells (Fig 5B). As seen with IR, all BARD1 variants and non-rescued cells depleted of BARD1 or BRCA1 were equally sensitive to cisplatin. In addition, BARD1 variant expression remained consistently equal to or greater than that of endogenous BARD1 in cells, indicating that decreased colony formation was not associated with decreased variant expression (Fig 5C). While decreased HDR function resulted in decreased colony formation, quantitative differences in the HDR activity did not correlate to quantifiable changes in sensitivity to cisplatin or IR.

## Discussion

In this study, we found: 1) from 10,389 cancer samples across 33 cancer types, 76 BARD1 missense variants were identified as potentially pathogenic and were selected for functional analysis. 2) 16 of the 76 tested variants were defective for HDR, suggesting that these were potentially pathogenic variants. 3) Four of the 17 variants tested in the ankyrin repeat domain, for which there was no previously known DNA repair function, were deficient in homologous recombination. 4) Five of the 19 variants tested in the BRCT domain, which does have known DNA repair functions, were deficient in homologous recombination. 5) Variants that were deficient in HDR rendered the cells sensitive to treatment with DNA-damaging cisplatin or IR. 6) Quantitative differences in HDR deficiency among defective variants did not translate to quantitatively different sensitivity to DNA damage.

The BRCA1-BARD1 heterodimer is necessary for tumor suppressor function [5,30]. Variants that affect binding between BRCA1 and BARD1 have been linked to familial breast cancer or are non-functional in the HDR assay [21,31,32]. Loss of BARD1 has been linked to increased susceptibility to hereditary breast and ovarian cancer (HBOC) and is associated with loss of tumor suppressor activity [4,13,33–36]. The importance of *BARD1* in cancer development indicates how significant it is to determine whether *BARD1* VUS are benign or pathogenic.

A rise in the quantity of genomic data has led to an increasing number of VUS, uncertain due to their low frequency and conflicting reports of pathogenicity. In this paper, potentially pathogenic *BARD1* variants were identified in a dataset of 10,389 cancer samples from 33 different cancers [22]. From germline and somatic samples, 76 variants from across the entire *BARD1* gene were identified as suggestive for being pathogenic. Analysis from the tumor sequencing data indicated BARD1 S339T, T343I, V523A, N450H, G451fs, and L239Q had significantly increased LOH, suggesting that these variants were more likely to be pathogenic. However, with the exception of the G451fs truncation variant, the other five variants were found to be functional in HDR. Previous work [19] has proposed that HDR-deficient BRCA1 variants could be identified using filtering based on increased LOH. In contrast, we have found that increased LOH does not correlate with HDR deficiency for BARD1 variants. As we are unaware of other studies regarding the relationship between LOH and HDR-deficient BRCA1 and BARD1 variants, these contrary results suggest that increased LOH is not a reliable indicator of non-functional variants. Data suggest that the HDR assay is a more effective method for identifying deficient variants. As the variant expression plasmids used only contain the mRNA coding sequence and we do not have access to patient samples, we cannot accurately examine the mRNA expression levels. However, if the mRNA expression levels of LOH mutants are lower than wild-type BARD1, this could indicate an HDR deficiency that we do not observe in our protein expression experiments.

In addition, the variant A460T, which was non-functional in HDR, was identified as potentially pathogenic during the original analysis of exome sequencing data [19] but was not considered pathogenic after updated analysis. These differences demonstrate the importance of empirical results, such as the HDR assay, for evaluating whether a given variant is predictive of cancer. Conversely, the results from functional assays may provide feedback for the improvement of the bioinformatic interpretation of genomic data. While large scale genomic analyses should be used to identify functionally significant variants, functional assays must still be utilized for more comprehensive characterization of these variants.

The 76 *BARD1* missense variants were tested in the HDR assay to examine DNA repair function. Variants were considered non-functional if their HDR activity was below 0.6 and significantly different from endogenous BARD1. Previous work in our lab [21] characterized fully non-functional variants as those with HDR activity significantly different from endogenous BARD1 but not empty vector, and intermediate variants as those significantly different from both endogenous BARD1 and empty vector. We have changed our classification standards based on the strength of the reproducibility in this study and for ease of analysis. We now interpret variants as functional or nonfunctional and do not include the intermediate phenotype. This interpretation is supported by the new data that the BARD1 A460T variant would have been ranked as intermediate using the previous interpretation, but assays measuring DNA damage sensitivity using IR and cisplatin indicated that BARD1 A460T was just as sensitive as the V767fs variant, which scored similar to the empty vector in the HDR assay.

Based on BRCA1 variant function in HDR, it was anticipated that BARD1 variants with impact on HDR function would map to the RING and BRCT domains. In this study, we tested eight variants in the BARD1 RING domain and none of them were defective for HDR. In a prior study [21], we had analyzed another nine variants in the BARD1 RING domain; three of these were defective for DNA repair activity. In the current study, we tested 19 variants in the BARD1 BRCT domain, and five of these were defective. BARD1 T598I, S660R, G698D, P707S, and G753D were found to be non-functional in the BRCT domain. The BRCT domain has been shown to interact with the HP1 protein in order to retain both the BRCA1-BARD1 complex and CtIP, which is involved in DNA end resection, at the damage site [26]. The BARD1-HP1 interaction is also necessary for the accumulation of DNA helicase FANCJ at sites of DNA damage [37]. BARD1 L570E/V571E and L570A/V571A variants have been shown to inhibit the interaction between BARD1 and HP1 [26]. The BARD1 BRCT domain is also necessary for binding poly(ADP-ribose) (PAR), allowing for rapid recruitment of the BRCA1-BARD1 complex to areas of DNA damage [27]. The variants K619A, C645R and V695L have been shown to disrupt BARD1-PAR interaction [27]. The T598 residue tested in this study (T598I) is located on the surface of the protein next to K619, and could possibly affect PAR binding. However, the relationship between BARD1-PAR binding and HDR is unclear since the BARD1 K619A and V695L variants, as well as several others that disrupt PAR binding, have been shown to be functional in HDR [38,39]. BRCA1 also binds to the BARD1 BRCT domain [40], and previous work in our lab has identified that this binding is affected by the BARD1 G623E variant [21]. Most of the non-functional variants we identified in the BRCT domain, with the exception of T598I, are not located near known binding sites for proteins associated with DNA repair.

To our surprise, we found four variants, BARD1 A460T, L465F, L480S, and P530L, were identified as non-functional in the ankyrin repeat domain. Prior to this study, the ankyrin repeat domain had no known reported function in DNA repair. Previous work has shown that a large deletion of the ankyrin repeat domain results in chromosome instability and loss of HDR function [38]. In addition, the BARD1 ankyrin domain has been shown to interact with p53 to mediate apoptosis [41]. The oncoprotein Bcl-3, which interacts with BARD1, is also involved in the regulation of NF-κB transcription via ankyrin repeat domain-associated protein interactions [42]. Our results indicate that the ankyrin repeat domain may have functions that are necessary for DNA repair. For both the ankyrin and BRCT domains, the non-functional variants identified may affect BARD1 folding and structure, which could also affect binding to proteins such as BRCA1 or HP1. For example, the BARD1 P707 and G753 residues are located near one another on the surface of the protein. As coding substitutions at these amino acids result in loss of HDR they may be part of a binding pocket. The identified variants may also indicate binding sites for proteins whose interaction with BARD1 has not yet been discovered. The characterization of non-functional *BARD1* variants in areas that are not well-studied helps to further understand the roles of the ankyrin repeat and BRCT domains in homology-directed repair.

Many of the *BARD1* variants tested have been recorded on ClinVar as having been isolated from patients with breast cancer or hereditary cancer-predisposing syndromes. Variants S241C and E361D, which were functional in HDR, have been identified has likely benign, and truncation variants V154fs, S551*, and Q564* are likely pathogenic. The variants V85L, R194K, I258T, N326S, R565H, and R641Q, which were functional in HDR, have conflicting reports of pathogenicity, with reports indicating they were VUS or likely benign. Since these variants were functional in the HDR assay, we would interpret such variants as likely benign. The non-functional truncating variant V767fs also had conflicting reports of pathogenicity, with reports indicating that it was a VUS or likely pathogenic. The trend observed is this paper is supplemented by the 29 variants that were previously studied [21]—variants V507M and R658C were functional in the HDR assay and are considered benign in ClinVar, and several other functional variants are listed as having conflicting reports of pathogenicity because reports indicate they are VUS or likely benign. Previous work has shown that pathogenic *BRCA1* variants are non-functional in the HDR assay, and benign variants are functional [16,17,19]. Based on the data from ClinVar, this trend appears to be true for *BARD1* variants as well, suggesting that the non-functional variants identified in this paper would be likely pathogenic.

We also asked whether BARD1 HDR function affected cell sensitivity to DNA damage agents. Cells expressing HDR-deficient variants A460T, P707S, G753D and V767fs, as well as endogenous-only, non-rescued cells depleted of BRCA1 or BARD1, were more sensitive to treatment with IR or cisplatin than cells expressing wild-type BARD1. Testing non-rescued cells allowed us to set a standard for the effect of non-functional DNA repair on damaged cells. We had hypothesized that the more HDR-deficient a variant was, the fewer colonies cells expressing that variant would form after damage, indicating a quantitative sensitivity to the DNA damage. The results did not support that expectation. Following treatment with IR or cisplatin, HDR-defective variants were in fact more sensitive to DNA damage, but they were as sensitive to DNA damage as cells depleted of BRCA1 or BARD1, suggesting that residual repair did not affect sensitivity.

The HDR assay indicates which variants are functional and non-functional, but it does not provide information on how this affects cell growth. This paper has shown that loss of homologous recombination results in increased sensitivity to DNA damage, as indicated by decreased colony formation. The HDR results also reveal a correlation between BARD1 variants that cause a loss of DNA repair function with those that are likely cancer predisposing. While we examined in this study a large number of *BARD1* variants across the length of the protein, including all three functional domains, many more *BARD1* VUS exist. In future work, we hope to mutagenize the *BARD1* functional domains on a larger scale, as we have previously done with the *BRCA1* N-terminus [29]. Creating a library of all potential *BARD1* variants in these functional domains and testing the HDR function of these variants would allow us to identify additional pathogenic variants and regions of interest. The work done in this study helps better understand the role of BARD1 in DNA repair, and how loss of homology-directed repair affects cell growth and sensitivity.

## Materials and methods

### Ethics statement

Sequencing results from de-identified tumors have already been published, and no additional ethical approval was required.

### Selection of BARD1 variants

BARD1 missense variants conferring potential cancer predisposition were identified in a cohort of 4,034 samples from 12 cancer types [19] that was part of larger set of 10,389 TCGA samples from 33 cancer types [22]. Germline single nucleotide variants (SNVs) were identified with variant calling on whole exome sequencing data using GATK [43] (version 3.5, using its haplotype caller in single-sample mode with duplicate and unmapped reads removed and retaining calls with a minimum quality threshold of 10) and VarScan [44] (version 2.3.8 with default parameters, except where–min-var-freq 0.10,–p value 0.10,–min-coverage 3,–strand-filter 1) operating on a mpileup stream produced by SAMtools (version 1.2 with default parameters, except where -q 1 -Q 13). Germline indels were identified using VarScan and GATK (same parameters and version as above) in single-sample mode. Pindel [45] (version 0.2.5b8 with default parameters, except where -x 4, -I, -B 0, and -M 3 and excluded centromere regions (genome.ucsc.edu)) was also applied for indel prediction. For all analyses, the GRCh37-lite reference was used and an insertion size of 500 was specified whenever this information was not provided in the BAM header.

All variants were limited to limited to coding regions of full-length transcripts obtained from Ensembl release 70 plus the additional two base pairs flanking each exon that cover splice donor/acceptor sites. SNVs were based on the union of raw GATK and VarScan calls, while indels were required to be called by at least two out of the three variant callers (GATK, VarScan, Pindel). High-confidence, Pindel-unique calls (at least 30x coverage and 20% VAF) were also included. Further, variants were required to have an Allelic Depth (AD) ≥ 5 for the alternative allele. Readcount analyses for variants passing these filters were performed in both normal and tumor samples using bam-readcount (version 0.8.0 commit 1b9c52c, with parameters -q 10, -b 15) in order to quantify the number of both reference and alternative alleles. Variants were required to have at least 5 counts of the alternative allele and an alternative allele frequency of at least 20%. Of these, rare variants were filtered for, with ≤ 0.05% minor allele frequency in 1000 Genomes and ExAC (release r0.3.1).

Variants passing manual review, with low allele frequencies (MAF < 0.05%), and significant LOH were prioritized for characterization. These variants, their cancer type distributions and frequencies are shown in the latest data release of the 10,389 samples [22] to the research community on NCI Genome Data Commons (https://gdc.cancer.gov/about-data/publications/PanCanAtlas-Germline-AWG).

### Cloning of *BARD1* variants

For transient expression, *BARD1* (NCBI Reference Sequence: NM 000465.3) wild-type and missense variants were cloned into a pcDNA3 vector backbone containing a rabbit *β*-globin intron upstream of the *BARD1* translation initiation site to drive expression of the 777 amino acid human BARD1 transgene. Variants were cloned using the New England BioLabs Q5 Site-Directed Mutagenesis kit. For stable integrations, *BARD1* wild-type and missense variants were cloned into a pcDNA5/FRT/TO vector backbone containing the His-Biotin-Tobacco Etch Virus (TEV) (HBT) tag [28]. PCR reactions were done using PfuUltra II Fusion HS DNA Polymerase. Vectors and inserts were ligated together using Gibson assembly [46]. Colonies with successful ligations were fully sequenced to confirm expected variants. All variants were verified using Sanger sequencing services provided OSU Comprehensive Cancer Center (OSUCCC) Genomics Shared Resource.

### Homology-Directed Repair (HDR) assay

For examining the HDR function of transiently expressed of *BARD1* variants, HeLa-DR-13-9 (HeLa-DR) cells were utilized. HeLa-DR cells contain two non-functional GFP coding sequences, one of which is interrupted by an I-SceI restriction endonuclease site. Cells were cultured in DMEM media containing 1% penicillin/streptomycin, 1% GlutaMAX, 10% bovine serum, and 1.5 μg/ml puromycin. Cells were seeded in a 24-well plate and transfected with siRNA to the *BARD1* 3’-UTR (5’-AGCUGAAUAUUAUACCAGAdTdT-3’) or control siRNA (5 pmol), and BARD1 wild-type, variant, or pcDNA3 empty vector (300 ng). All transfections were carried out using Lipofectamine 2000 per the manufacturer’s recommendations. Cells were moved to 6-well plates 24 hours later. 48 hours after the first transfection, cells were transfected with 25 pmol siRNA, 750 ng DNA, and 750 ng of expression plasmid containing the restriction endonuclease I-SceI to induce a double-strand break. If HDR is functional, the break is repaired by gene conversion with the second GFP allele, and cells become GFP-positive [16,25]. 72 hours after the second transfection, cells were collected and GFP-positive cells were counted using the FACSCalibur in the OSUCCC Analytical Cytometry Shared Resource. 10,000 cells were counted, and the remaining cells were used for immunoblotting. Cells transfected with *BARD1* siRNA and BARD1 wild-type plasmid (wild-type rescue), and cells treated with control siRNA and empty vector, served as positive controls. Cells treated with *BARD1* siRNA and empty vector were used as a negative control. HDR activity, as defined by the percentage of GFP-positive cells, was normalized to wild-type rescue control and set to 1.

For examining the HDR function of stably integrated *BARD1* variants, HeLaDR-FRT/TR cells [29] were used. Cells integrated with pcDNA5-FRT/TO-HBT-tagged *BARD1* wild-type or variants (A460T, P707S, G753D and V767fs) were seeded in 24-well plates. Cells not integrated with *BARD1* variants were used as a negative control. Cells were transfected with siRNA to the *BARD1* 3’-UTR or control siRNA. All transfections were carried out using Oligofectamine according to the manufacturer’s recommendations. Transfections were carried out on the same time pattern as detailed for transiently expressed *BARD1*. For 24-well transfections, 30 pmol siRNA was used, and for 6-well transfections, 50 pmol siRNA and 3 μg of I-SceI expression plasmid were used. Cells were collected and GFP-positive cells counted as detailed for transiently expressed *BARD1*. HDR activity, as defined by the percentage of GFP-positive cells, was normalized to cells treated with control siRNA for each individual cell line and set at 1.

### Alignment of BARD1 protein sequences

*Homo sapiens*, *Felis catus*, *Canis lupis familiaris*, *Mus musculus*, *Monodelphis domestica*, and *Ovis aries* BARD1 protein sequences were aligned using Clustal Omega [47] to examine conserved residues.

### Integration of variants into HeLaDR-FRT/TR cells

pcDNA5-FRT/TO-HBT-tagged *BARD1* wild-type and variants were co-transfected with plasmid expressing the flippase recombinase in a 1:2 ratio into HeLa-DR-FRT/TR cells to induce integration at the flippase recognition target site [48,49]. Transfections were done according to Lipofectamine 2000 manufacturer’s recommendations. 24 hours after transfection, cells were incubated at 30°C for 24 hours and then moved back to 37°C. Integrated cells were selected for with 550 μg/ml Hygromycin-B.

### Immunoblotting

For *BARD1* variants, replicates were combined, spun down at 1200 rpm for 5 minutes and resuspended in 150 μl of 1X LDS-PAGE dye. Samples were sonicated at 45% for 15 seconds three times. Sample was resolved on 6 or 8% SDS-PAGE gels and transferred to PVDF membrane. Samples were probed with BARD1 (Bethyl, 1:1000) and BRCA1 (1:500) [50] antibodies. Antibodies to RHA1 (1:20000) [50] and α-tubulin (Sigma, 1:20000) were used as loading controls. Membranes were incubated with fluorescent (LI-COR, 1:20000) or chemiluminescent (GE, 1:5000) rabbit and mouse secondary antibodies.

### Clonogenic assays

HeLaDR-FRT/TR cells stably expressing pcDNA5-HBT-BARD1 WT, A460T, P707S, G753D and V767fs, as well as control cells expressing only endogenous BARD1, were seeded in 24-well dishes and transfected with 30 pmol of siRNA to the *BARD1* 3’-UTR, *BRCA1* 3’-UTR or control siRNA. Transfections were done using Oligofectamine as per the manufacturer’s protocol. Cells were transferred to 6-well dishes after 24 hours, and were treated with 50 pmol siRNA 48 hours after the first transfection. 48 hours after the second transfection, 1000 cells of each treatment condition were plated in 10-cm dishes. Remaining cells were saved for immunoblotting to confirm knockdown. After 24 hours, cells were treated with either ionizing radiation (IR) or cisplatin. Cells that were treated with IR were subjected to 0, 1, 2, 4, or 6 Gy using the RS 2000 X-Ray Irradiator. For cisplatin treatment, cells were treated with 0, 1,875, 3.75, 7.5, and 15 μM of cisplatin for 2 hours, after which cells were washed twice with 1X PBS and fresh media was added. Untreated cells were used as a control. After two weeks of growth at 37°C, cells were fixed with cold methanol and stained with crystal violet. Dishes were coded to blind their treatment and cells were counted using OpenCFU [51]. The log value of the count was used for comparison.

### Statistical analysis

All *BARD1* variants in the HDR and clonogenic assays were tested in triplicate. For HDR assays using transiently expressed *BARD1* variants, HDR activity was normalized to wild-type rescue, which was set to 1. The Student’s *t*-test was applied to determine whether BARD1 variant HDR activity significantly differed (p < 0.01) from endogenous BARD1. Variants that were significantly different and below the cutoff of 0.6 were considered non-functional. For clonogenic assays, the Student’s *t*-test was carried out to examine whether BARD1 variant-expressing and endogenous-only cells treated with *BARD1* or *BRCA1* 3’-UTR siRNA formed a significantly different number of colonies than variants treated with control siRNA and cells expressing BARD1 wild-type (p < 0.05).

## Supporting information

S1 Table GFP expression percentages for BARD1 variant HDR assays. (XLSX)

S1 Fig Functional analysis of 105 *BARD1* variants. 105 *BARD1* single missense substitutions were tested for function in the HDR assay. Results from Lee at al. 2015 are included with variants tested in this paper. HeLa-DR cells [16] were treated with control siRNA (lane 1) or siRNA specific to the *BARD1* 3'-untranslated region (UTR) (lanes 2–109) and empty vector (lanes 1, 2) or *BARD1* expression plasmid (lanes 3–109). Two positive controls were used: cells treated with empty vector and control siRNA (lane 1), and cells depleted of endogenous BARD1 with wild-type BARD1 rescue (lane 3). Cells treated with empty plasmid and *BARD1* 3'UTR siRNA were used as a negative control (lane 2). HDR function was characterized by the percentage of GFP-positive cells measured using flow cytometry. Results in each experiment (±S.E.M.) were normalized to the WT rescue (lane 3), which was set equal to 1. Results represent three independent transfections per *BARD1* expression plasmid. Variants that are benign and pathogenic according to ClinVar are labeled blue and red respectively. Variants with conflicting interpretations are labeled gray. HDR-deficient variants are marked by an asterisk and classified by having HDR function less than 0.6 and p < 0.01 when compared to endogenous BARD1 (control siRNA) using the Student’s *t*-test.
(TIF)

S2 Fig BARD1 protein sequence differences between mammalian species. Human (Homo_sapiens), domestic cat (Felis_catus), domestic dog (Canis_lupis_familiaris), mouse (Mus_musculus), gray short-tailed opossum (Monodelphis_domestica) and domestic sheep (Ovis_aries) BARD1 protein sequences were aligned using Clustal Omega to examine conserved residues. Asterisks (*) indicate positions with a single, fully conserved residue. Colons (:) indicate residue conservation between groups of strongly similar properties. Periods (.) indicate conservation between groups of weakly similar properties. Residues that were mutated in HDR-deficient, non-truncating variants are highlighted in yellow. Residues mutated in HDR-functional variants are highlighted in green.
(PDF)

S3 Fig Immunoblots of truncated BARD1 variants. Truncated BARD1 variants tested in the HDR assay were examined for their expression relative to endogenous BARD1. Replicates were pooled together to examine BARD1 expression. The BARD1 protein is indicated with an arrow. The endogenously expressed BARD1 in control transfections (lanes 1, 10) and BARD1 WT rescue (lanes 3, 12) were compared with the expression of truncated BARD1 variant proteins. Variants were run on 4–12% Bis-Tris and 6% acrylamide gels due to a contaminating band that co-migrated with BARD1 WT on 4–12% Bis-Tris gels. Variants G451fs (lanes 6, 15), S551* (lanes 7, 16), Q564* (lanes 8, 17) and V767fs (9, 18) expressed truncated protein. Variant V154fs (lanes 4, 13) does not contain the residue recognized by the BARD1 antibody used, and may express truncated protein.
(TIF)
