# Supplementary figures and images for: Functional analysis of BARD1 missense variants in homology-directed repair and damage sensitivity
Source: PLoS Genet. 2019 Mar 29;15(3):e1008049. doi: 10.1371/journal.pgen.1008049 (PMC6457558; doi:10.1371/journal.pgen.1008049)

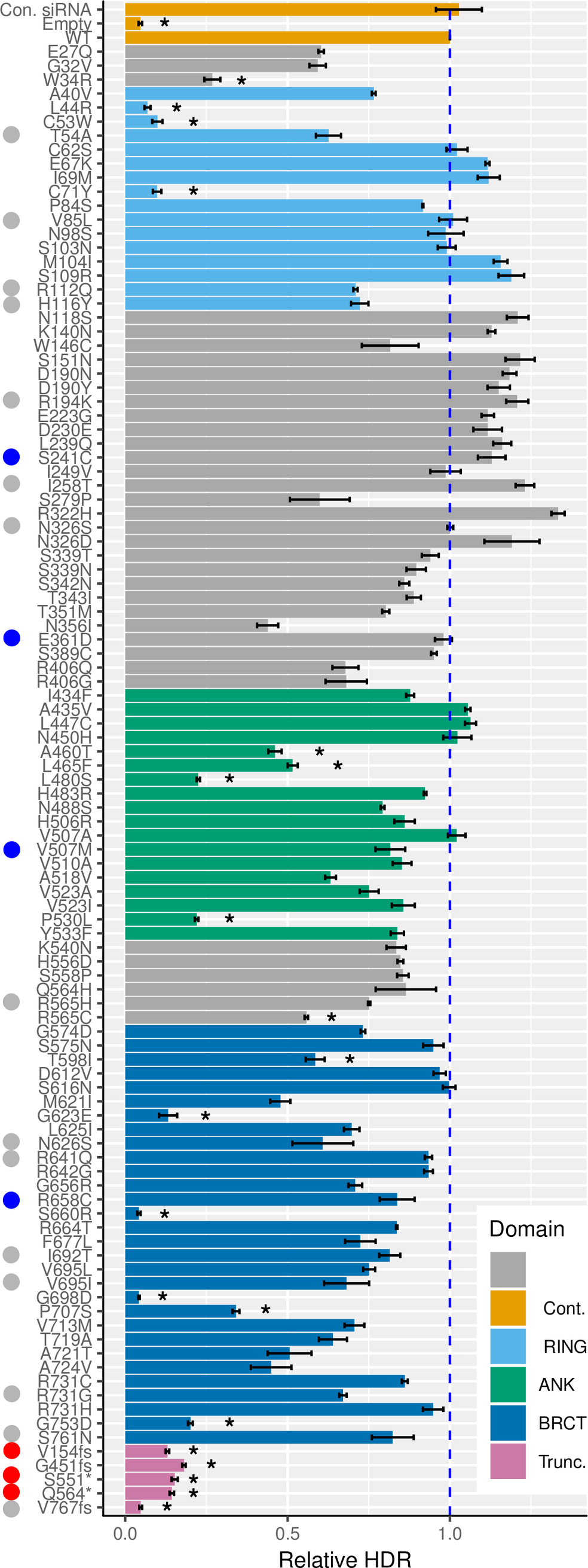

Supplement: S1 Fig — 105 BARD1 single missense substitutions were tested for function in the HDR assay. Results from Lee at al. 2015 are included with variants tested in this paper. HeLa-DR cells [16] were treated with control siRNA (lane 1) or siRNA specific to the BARD1 3'-untranslated region (UTR) (lanes 2–109) and empty vector (lanes 1, 2) or BARD1 expression plasmid (lanes 3–109). Two positive controls were used: cells treated with empty vector and control siRNA (lane 1), and cells depleted of endogenous BARD1 with wild-type BARD1 rescue (lane 3). Cells treated with empty plasmid and BARD1 3'UTR siRNA were used as a negative control (lane 2). HDR function was characterized by the percentage of GFP-positive cells measured using flow cytometry. Results in each experiment (±S.E.M.) were normalized to the WT rescue (lane 3), which was set equal to 1. Results represent three independent transfections per BARD1 expression plasmid. Variants that are benign and pathogenic according to ClinVar are labeled blue and red respectively. Variants with conflicting interpretations are labeled gray. HDR-deficient variants are marked by an asterisk and classified by having HDR function less than 0.6 and p < 0.01 when compared to endogenous BARD1 (control siRNA) using the Student’s t-test. (TIF) [file pgen.1008049.s002.tif]

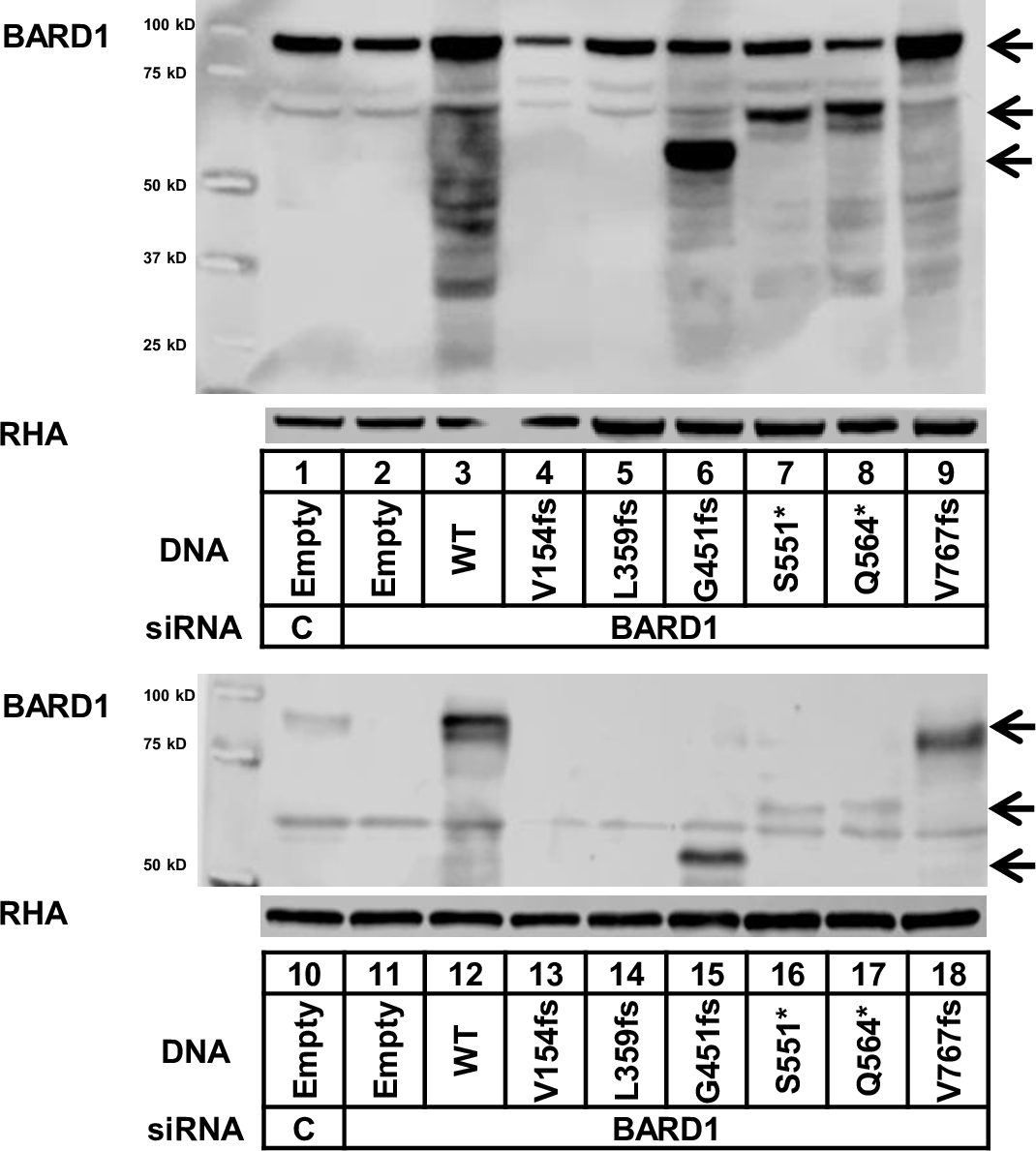

Supplement: S3 Fig — Truncated BARD1 variants tested in the HDR assay were examined for their expression relative to endogenous BARD1. Replicates were pooled together to examine BARD1 expression. The BARD1 protein is indicated with an arrow. The endogenously expressed BARD1 in control transfections (lanes 1, 10) and BARD1 WT rescue (lanes 3, 12) were compared with the expression of truncated BARD1 variant proteins. Variants were run on 4–12% Bis-Tris and 6% acrylamide gels due to a contaminating band that co-migrated with BARD1 WT on 4–12% Bis-Tris gels. Variants G451fs (lanes 6, 15), S551* (lanes 7, 16), Q564* (lanes 8, 17) and V767fs (9, 18) expressed truncated protein. Variant V154fs (lanes 4, 13) does not contain the residue recognized by the BARD1 antibody used, and may express truncated protein. (TIF) [file pgen.1008049.s004.tif]
